# A curated genome-scale metabolic model of *Bordetella pertussis* metabolism

**DOI:** 10.1371/journal.pcbi.1005639

**Published:** 2017-07-17

**Authors:** Nick Fyson, Jerry King, Thomas Belcher, Andrew Preston, Caroline Colijn

**Affiliations:** 1 Department of Mathematics, Imperial College, London, UK; 2 The Milner Centre for Evolution and Department of Biology and Biochemistry, University of Bath, Bath, UK; Centre for Research and Technology-Hellas, GREECE

## Abstract

The Gram-negative bacterium *Bordetella pertussis* is the causative agent of whooping cough, a serious respiratory infection causing hundreds of thousands of deaths annually worldwide. There are effective vaccines, but their production requires growing large quantities of *B. pertussis*. Unfortunately, *B. pertussis* has relatively slow growth in culture, with low biomass yields and variable growth characteristics. *B. pertussis* also requires a relatively expensive growth medium. We present a new, curated flux balance analysis-based model of *B. pertussis* metabolism. We enhance the model with an experimentally-determined biomass objective function, and we perform extensive manual curation. We test the model’s predictions with a genome-wide screen for essential genes using a transposon-directed insertional sequencing (TraDIS) approach. We test its predictions of growth for different carbon sources in the medium. The model predicts essentiality with an accuracy of 83% and correctly predicts improvements in growth under increased glutamate:fumarate ratios. We provide the model in SBML format, along with gene essentiality predictions.

## Author summary

Metabolic flux models have been used to understand how organisms adapt their metabolism under different growth conditions, and are finding increasing application in synthetic biology and biotechnology. One barrier to progress in this field is the construction and curation of metabolic flux models for new organisms. Here we present a curated genome-scale metabolic flux model for *Bordetella pertussis*, the causative agent of whooping cough. Producing vaccines against whooping cough requires growing *B. pertussis* in large volumes. However, its growth is relatively slow, final yields of biomass are relatively low and growth characteristics can be variable. Understanding *B. pertussis* metabolism has applications to improving vaccine production, as well as in understanding the basic biology of this organism.

## Introduction

*B. pertussis* is a Gram-negative bacterium that causes whooping cough, a respiratory infection responsible for significant annual mortality worldwide [1, 2], especially among infants and young children. *B. pertussis* is described as a fastidious organism. It does not metabolise sugars as carbon source as it does not possess an intact glycolysis pathway [3]. Amino acids appear to be the primary carbon sources for growth. *B. pertussis* can grow using most of the amino acids as a carbon source, however alanine, proline and glutamate are utilized preferentially suggesting that amino acids that are degraded to *α*-ketoglutarate or pyruvate are oxidized rapidly. Several studies have demonstrated that glutamate is by far the most efficiently metabolized and is considered to be the main carbon source for growth of *B. pertussis* [3–5], which can be grown in the lab using solely glutamate as a carbon source and cysteine as a source of sulphur (along with salts and some vitamins).

It was a long-held view that the TCA cycle was not completely functional in *B. pertussis*. This stemmed from the inability of *B. pertussis* to utilise citrate as a carbon source along with observations of the build up of poly-hydroxybutyrate and release of free fatty acids in batch cultures. However, the *B. pertussis* genome contains genes that appear to encode a complete pathway [6]. Recently, demonstration of citrate synthase, aconitase and isocitrate dehydrogenase activities in *B. pertussis* gave a clear indication that the TCA cycle is fully functional, although it remains unclear why citrate does not support *B. pertussis* growth [7].

Commonly used media for broth growth, such as Stainer-Scholte (SS) broth [8], contain glutamate as the main carbon source. Modified SS broth contains casamino acids and heptakis, and growth is enhanced by these additions. Casamino acids probably increase the level of glutamate and enable utilization of other amino acids. Heptakis, a cyclodextrin, absorbs free fatty acids that are inhibitory towards *B. pertussis* growth [9]. However, culture of *B. pertussis* in SS broth leads to an imbalance in N:C ratios leading to the formation of ammonium which is inhibitory to growth, resulting in relatively low final cell densities.

Several studies have investigated parameters affecting the growth rate of *B. pertussis* using either batch cultures or steady state cultures in bioreactors (for example see [3, 5, 10, 11]). These informative studies revealed much of what is known about *B. pertussis* growth parameters, identifying the importance of balancing N:C ratios, avoiding excessively high substrate concentrations and the effect of salt concentrations for attaining high biomass yields.

The slow growth and limited yields of *B. pertussis* in culture are important limitations to the efficiency of *B. pertussis* vaccine production. In particular, at least five times more culture volume is required to generate one dose of an acellular pertussis vaccine compared to a whole cell one. Expansion of *B. pertussis* vaccination programmes using acellular vaccines, either into the developing world that for the most part use whole cell vaccines, or to increase the use of booster doses for adolescents/adults would place strain on global production of these vaccines. Increased efficiency of *B. pertussis* culture would help to alleviate these strains but this requires greater knowledge of the growth characteristics of *B. pertussis*.

Flux balance analysis (FBA) is an established approach for modelling the metabolic networks of organisms at the genome scale, and is a framework for integrating other ‘omics data layers with metabolism [12–16]. Briefly, the network of metabolic reactions in an organism is represented by an *m* × *n* stoichiometric matrix, *S*. Each row of *S* represents a metabolite and each column gives the stoichiometry for a particular metabolic reaction. There are *m* metabolites and *n* reactions. The list of metabolites includes both so-called “internal metabolites”, which are not exchanged with the growth medium or environment, and “external metabolites”, which are. External metabolites include nutrients in the modelled growth medium, metabolites that diffuse in and out of the cell, and by-products of growth that leave the cell. FBA models make the approximation that the time scale of interest (hours or longer) is long enough that short-term transients in the kinetics of individual reactions (which would usually dissipate in seconds or minutes) will have largely passed, so that reactions are running at steady state: there is no net production or consumption of (internal) metabolites. Mathematically, each reaction is associated with a flux *v*; the steady-state approximation is the constraint *Sv* = 0. The specific growth medium and uptake rates mean that there are constraints on how fast the influx of nutrients can be; mathematically, this means that there are constraints on some or all of the reaction fluxes. Finally, FBA models describe the growth capacity of an organism using an objective function *c*: how much of the given objective could the metabolic network possibly produce, at steady state, under the given constraints? The objective is typically a biomass vector, *c*, describing the major components of the dry weight of the cells. FBA models then approximate the metabolic network’s capacity to produce this biomass under various conditions. FBA is performed by solving a linear programming problem:

$$\begin{array}{ccc} \max & c\cdot v & \\ s.t.\;\;\;Sv & = & 0\\ a_{r}\leq & v_{r} & \leq b_{r}. \end{array}$$ (1)

where *S* is the stoichiometic matrix, *v* is a vector of reaction fluxes, *c* is the objective function, and *a*_*r*_ and *b*_*r*_ are vectors of length *n* describing lower and upper constraints on the reaction fluxes. A growth medium is defined by setting constraints so as not to allow uptake of nutrients that are not present in the medium.

In principle, an FBA model can be constructed directly from an annotated genome; where a gene’s enzymatic function is known, the relevant reaction and metabolites can be added to the system and the stoichiometric matrix can be constructed so as to capture the (usually conserved) stoichiometries of the included reactions. In practice, genome annotation and functional prediction is imperfect, and FBA models require substantial curation [17]. This typically requires first constructing a draft FBA model based on the annotation in an automated way, then examining each reaction in *S* and determining whether it describes realistic biochemistry, as well as examining the gene(s) associated with it, their annotation in the organism and whether the gene-reaction relationship is appropriate. This process requires considerable knowledge of the organism’s biochemistry, and is labour intensive [17–20].

Previously, dynamic models of limited compartments of *B. pertussis* were developed and demonstrated the utility of this approach for interrogating specific facets of *B. pertussis* metabolism. Here, we present the first published genome-scale metabolic reconstruction for *B. pertussis*. It is suited for flux balance analysis, and models *B. pertussis*’ metabolic reactions accordingly. We refer to this reconstruction as “the model” or “metabolic model” throughout. To demonstrate the use of the model to interrogate *B. pertussis* growth, we used it to predict reactions that are essential for growth on laboratory medium. We tested these predictions by performing a genome-wide screen for essential genes using a Transposon-directed Insertional Sequencing (TraDIS) approach [21] and demonstrate a high degree of concordance between model predictions and experimental observations. We used the model to investigate the reduction of ammonia production that occurs during growth in standard medium, and tested the predictions arising. The development of a genome-scale model provides a valuable tool for investigating the growth of this bacterium.

## Methods

Recent advances in theory and computational power have allowed increasing automation in reconstructing full genome metabolic models. While still requiring considerable manual work to refine them, draft models can be produced rapidly and easily from annotated genomes. We used the Model Seed framework as the starting point for our model. The Model SEED integrates a range of existing approaches into a coherent pipeline, accessed through a web interface [18, 22].

Our initial model was obtained from the SEED interface, uploading the genome sequence of the Tohama I strain of *B. pertussis* (Genbank accession number NC_002929.2). The genome sequence was then reannotated by the integrated RAST annotation servers, before this annotated version was used in the reconstruction of the model. The process is fully automated, undertaking a series of steps to ensure the resulting model is capable of producing the specified biomass vector under FBA simulation. Details of the steps in the Model SEED reconstruction are discussed below as pertinent to the steps in our manual curation, and full details can be found in the paper by Henry et al. [18].

### Curation of the metabolic model

#### Biomass objective function

We performed experiments to determine the composition of *B. pertussis* biomass and used the results to define a biomass objective function for the model. The biomass objective function (BOF) is a special reaction in FBA which defines key biomass components, in specified ratios, that a metabolic network must produce in order for the bacteria to grow. By default, the Model SEED produces an organism-specific template biomass function, based on near-complete BOFs for all the organisms examined in their original study. The template reaction includes all universal biomass components. It also includes non-universal components but only if criteria are satisfied that specify the metabolic subsytems and functional roles a genome must contain for the component to be added to the biomass reaction template [18]. Inevitably this will not provide perfect results for new organisms, but provides a solid starting point for organism-specific manual curation. Our model uses SEED default single reactions to denote protein, RNA and DNA synthesis. We adjusted the stoichiometry of the major biomass components to reflect our experimental data. We also examined all components of the automatically-created biomass objective to eliminate any obvious mistakes, including spurious metabolites that were (incorrectly) required components of any growth medium simply due to their presence in the objective. An ATP cost is incorporated into the BOF to reflect the ATP costs of diverse cellular functions that are necessary for growth but are not explicitly included in the model.

#### Biomass composition assays

*B. pertussis* strain BP536 was used in these studies. BP536 is a streptomycin resistant derivative of the genome sequence strain Tohama I; they contain the same metabolic gene repertoire and metabolic network. Bacteria were grown in 100 mls of SS broth, supplemented with heptakis at 1g/L, for 48 hours. The OD_600_ of the cultures were recorded and the bacteria were pelleted by centrifugation in a microfuge from an appropriate volume of culture to give the equivalent cells for 20mLs of an OD_600_ = 1.0. These cells were freeze dried in preweighed tubes to enable measurement of the dry weight of cells in this culture volume. Appropriate volumes of culture were processed for measurement of DNA, RNA, protein, lipid and carbohydrate. Genomic DNA was extracted using a GenElute Genomic DNA kit (Sigma, Poole, UK). DNA was eluted using seven elution steps with 200*μ*L of water each time. Optimisation trials demonstrated that DNA was eluted for up to 7 elution steps. Eluate was collected in a preweighed tube to enable accurate measurement of the total elution volume. RNA was extracted using stabilization with RNAprotect Bacteria reagent (Qiagen Ltd, Manchester, U.K.) and extraction using the RNeasy extraction kit (Qiagen Ltd). RNA was eluted using 250*μ*L of water into preweighed tubes to allow accurate measurement of eluate volume. DNA and RNA were quantitated using a Qubit (Life Technologies Inc, Paisley, U.K.). Protein was measured using an assay based on the Bradford assay: the Bio-Rad Protein Assay (Bio-Rad Laboratories, Hemel Hempstead, UK) using BSA as the protein standard. Carbohydrate was measured using a phenol-sulphuric acid assay using glucose as the standard [23]. Total lipid was extracted using the Bligh-Dyer method [24]. The measurements were converted to percentage of dry cell weight. Triplicate samples were processed for each assay, and the experiment repeated three times.

#### Gap filling

The SEED algorithm [18] engages in automated filling of gaps in pathways, systematically plugging holes until a viable model is achieved. The pipeline adds the minimal number of missing reactions required to fill gaps that prevent synthesis of the specified biomass components. The added reactions are selected from a database that comprises all of the biochemistry represented by the KEGG database. We inspected each gap-filled reaction in the preliminary model. In the case of *B. pertussis* these checks are particularly important, since it has evolved through a process of genome reduction meaning that remnants of pathways may be encoded by the genome [6].

#### Known growth conditions

Part of the process of model curation is to ensure it can recreate known behaviour. We curated the model to ensure that its ability to grow on previously-defined media was correct. We compiled data on viable and non-viable growth media formulations [3, 4, 8, 25]. We then simulated growth on these media by specifying the exchange reaction constraints to model the media, and using FBA to determine whether the model could produce biomass under those constraints. Where necessary, the model was adjusted to bring it in line with experimental results. In some cases this involved corrections to the exchange reactions present in the model, and in others the removal of key reactions to disable pathways that made spurious use of metabolites such as glucose.

#### Thermodynamic viability

Electrons, in the form of H- hydride ions, are passed along the electron transport chain in a particular sequence. Although in principle every step of the chain is reversible, in practice problems arise if it is allowed to run in both directions. Energetically unfavourable reaction sequences are able to occur, leading to the possibility of free ATP production. These manifest themselves as sets of reactions that ‘freewheel’, feeding into one another and running at a rate much higher than the baseline level for the particular flux state. These loops can provide free sources of energy to the cell, and hence must be removed in order to ensure realistic results. As addressed in Thiele et al. [26], problems with the electron transport chain can often be traced back to reactions that use quinones as electron receptors. We examined all such reactions, and where necessary we altered their direction and reversibility to control any thermodynamically infeasible loops.

#### KEGG associations

The SEED Model included associations between reactions in the model and KEGG reactions. We manually examined the KEGG associations and used homology searches using BLAST algorithms to update associations. A number of these appeared to be associating different reactions to the same enzyme, for example similar reactions but operating in different pathways. These wrong associations arose from the annotation ascribing an incorrect (or too specific) EC number to an enzyme, or from misannotation of enzyme function. In total 170 KEGG associations were corrected (S1 Table).

#### Pseudogenes

Pseudogenes are genes that have suffered a disabling mutation (for example a single nucleotide mutation that introduces a premature stop codon, or a single base pair deletion that causes a frame-shift mutation) rendering the gene non-functional; this has occurred recently enough for the coding sequence to be unchanged from the functional version except for the mutation. *B. pertussis* is noteworthy for containing an unusually high number (358) of pseudogenes arising during its evolution via genome reduction (Parkhill et al, 2003). Many genome annotations contain the full amino acid sequence of the pseudogene and the SEED model included these as functional enzymes. We removed reactions assigned to pseudogenes from the model.

#### Maintenance ATP costs

The model allows for both growth-associated (GAM) and non-growth-associated (NGAM) maintenance ATP costs, to capture the energy use of processes that are not explicitly described in the model [26, 27]. In our gene essentiality computations we model the GAM as a flux of 40 units ATP per unit biomass and we do not require an NGAM ATP flux. Gene essentiality results are unmodified if the NGAM is constant; as ATP is required for biomass growth, its requirement in the NGAM does not change whether reactions are deemed essential. In principle, the ATP costs can be calculated experimentally by measuring substrate uptake as a function of growth rate, usually done using chemostat cultures. This approach has proved particularly difficult for *B. pertussis*, as the growth rate is relatively insensitive to carbon source concentrations, with final yield of biomass varying rather than growth rate, for example [3, 11]. This suggests that uncharacterized regulatory mechanisms, or non-metabolic control, are operating. Thus, the ATP cost used here, which is standard in other metabolic models, is a sensible compromise.

### Experimental determination of essential genes

Essential genes were identified using Transposon Directed Insertion-Site Sequencing (TraDIS) [21]. Saturated transposon libraries were constructed using the pBAM1 delivery vector [28], modified with PmeI restriction sites for digestion of vector-derived amplicons prior to sequencing. The details of construction of the transposon library, sequencing of insertion sites and analysis of insertion site frequency followed the approaches described previously for TraDIS [29]. Three independent transposon libraries were made. Each were plated on charcoal agar (Oxoid) supplemented with 50*μ*g/mL kanamycin and incubated at 37°C for 72 hours. Between 300 000 and 500 000 transposon mutants were harvested per library and processed for TraDIS. Insertion indexes were calculated for each gene and essentiality calculated using the cut off point described previously [21].

#### In silico essentiality predictions

The FBA modelling approach allows the manipulation of a metabolic network of the cell to make predictions about what impact these interventions will have on growth rate. In particular, the link between reactions in the model and their catalysing proteins can be used to predict which genes will be fatal if knocked out from the genome. Comparing predictions about essentiality from the model with experimental essentiality data from real-world experiments allows a valuable test of the model [18]. This test was performed with the *B. pertussis* model.

We simulated growth of the *B. pertussis* model on a rich growth medium, modelled on the charcoal agar used in the TraDIS experiment. To simulate the protein-rich growth environment we enhanced the minimal medium based on glutamate growth with free uptake of the full range of amino acids. We then ran through each gene in turn and removed all reactions from the model where annotations indicated the gene was required to catalyse the reaction. Optimizing for the biomass objective function, we ran FBA and normalised the achieved flux (*c* ⋅ *v*), dividing by that seen in the unmodified (wild type; all reactions present) network. This resulted in a relative growth rate for each knockout, ranging from 1 (indicating that the gene’s removal has no impact on growth) to 0 (removal of this gene meant there were no feasible solutions to the optimization; growth not possible).

Results from the TraDIS experiment represent the best indicator of gene essentiality in *B. pertussis*, and in assessing the quality of the model’s performance, we treat this data as the “ground truth”. Our computational modelling of essentiality can be viewed as a classifier, giving each gene a score as to how essential it is. To convert this into a binary essential/non-essential classification, we need to choose a threshold on the relative growth rate. The standard approach to assessing performance of such classifiers is the Receiver Operating Characteristic (ROC) curve. If the threshold is smoothly varied, we can calculate the True Positive Rate (TPR) and False Positive Rate (FPR) for each point, and these are then plotted. The total area under this curve is an indication of the performance of the classifier.

#### Measuring ammonia production during growth on different glutamate:fumarate ratios

Media were prepared containing different ratios of glutamate:fumarate (5:1, 2:1, 1:2 and 1:5) in terms of contribution of carbon atoms. SS broth was used as the basal media for this. Plate grown *B. pertussis* were resuspended in SS broth. This suspension was used to seed two 30mls cultures in 250mls flasks. These were grown for 24 hours at 37°C with shaking. At this point cells were pelleted by centrifugation and washed in PBS. Cells were then resuspended in the various media at an OD600 of 0.1. Ten wells of 250uls of suspension in each media were seeded into a round bottom 96 well plate and grown at 37°C with shaking in a Fluostar Omega plate reader (BMG Labtech, Aylesbury, UK) until stationary phase was reached. The OD600 was measured every 15 minutes. At the end of growth, the culture was removed from five of the wells for each medium and the bacteria were pelleted in a microcentrifuge. The supernatant was removed and stored at -80°C. The concentration of ammonium in the supernatants was measured using an assay kit (Product number AB83360, Abcam, Cambridge, UK) as described in the manufacturer’s protocol.

## Results

The final model consists of 1152 reactions and 1191 metabolites, which are described in Table 1. We note that the raw (non-curated) model was unable to generate biomass when the components of standard growth medium for *B. pertussis* (SS broth) were used to specify the available exchange reactions.

**Table 1:** Breakdown of the reactions and metabolites found in both the original and curated model. The curation process involved both the removal and addition of elements in the model, and we show how the final set of reactions and metabolite break down into categories within the cell.

	Reactions	Metabolites
Initial	1203	1143
Removed	110	3
Added	59	54
Exchange	99	-
Transport	72	-
Cytoplasm	-	993
External	-	99
Boundary	-	99
Blocked	199	301
Final	1152	1191

### Curation of the preliminary model

Extensive curation of the preliminary model was performed. Key changes are discussed below. The initial ModelSEED model file and a detailed curation history file are included as supplemental files to allow specific aspects of our curation and the effects of alternative curations to be investigated.

### Gap-filled reactions

Reactions that were automatically gap-filled were analysed. Based on known behaviours of *B. pertussis*, gap-filled reactions were removed to create true gaps (e.g. nicotinate, cysteine auxotrophy [30]), removed as the reactions do not occur in *B. pertussis* (e.g. 11 reactions specific to synthesis of *E. coli* rather than *B. pertussis* LPS), or genes identified that encode the probably missing function. This process left only 10 gap-filled reactions for which no gene assignment exists. These reactions are listed in S2 Table.

### Pyridoxal phosphate (PLP) biosynthesis

PLP is an essential cofactor. The SEED model included two gap-filled enzymes corresponding to the PdxT/PdxS catalysed generation of PLP from glyceraldehyde-3-phosphate and ribulose-5-phosphate, as characterised in *B. subtilis*. However, there are no homologs of *pdxT* or *pdxS* in *B. pertussis*. An alternative well characterised pathway for PLP synthesis can occur via the activities of PdxB, PdxA and PdxJ. Clear homologs of both *pdxA* and *pdxJ* are evident in *B. pertussis*. PdxB is 4-phosphoerythronate dehydrogenase, an oxido-reductase enzyme. These enzymes generally show low levels of sequence conservation between homologs. Using BlastP of the *E. coli* PdxB sequence against the *B. pertussis* genome identified 4 putative dehydrogenases with scores in the range of 3e-10 to 5e-15. Thus, it was concluded that there are potential PdxB candidates in *B. pertussis* and as PLP synthesis is expected to be essential, gap-filling of the PdxB-catalysed reaction was more logical than that of the PdxT/PdxS reaction.

### Quinolinate synthase

The SEED model filled gaps in the reactions catalyzed by quinolinate synthase, encoded by *nadA* and L-aspartate oxidase, encoded by *nadB*. There are no clear homologs of *nadA* or *nadB* in *B. pertussis* and this bacterium is auxotrophic for nicotinate, which is a component of the *B. pertussis* growth media. Thus, it is expected that the nicotinate synthesis pathway is incomplete in *B. pertussis*. These reactions were changed to true gaps in the model.

### Protoporphyrinogen-IXoxygen oxidoreductase

The reaction catalyzed by this enzyme is a critical step in the synthesis of the cofactor heme. In *B. pertussis* there are no identifiable homologs of genes encoding the HemG or HemY members of this family of enzymes, although the remainder of the pathway appears to be present. In some other bacteria missing HemG/Y an alternative gene, *hemJ*, encodes this activity. BP2372 was identified as a potential *hemJ* homologue and was not associated with any other reaction in the model. Thus, the model was curated to include BP2372 as performing this step.

### Thiamine phosphate biosynthesis

Thiamine phosphate is a crucial cofactor. The SEED model contained thiamine phosphate biosynthesis based on the pathways described in *E. coli* in which ThiH catalyses the production of 4-hydroxy-benzylalcohol from tyrosine. However, in the model, 4-hydroxy-benzylalcohol is a dead-end metabolite, as it is not used in any pathway and the model constrains all fluxes producing dead-end metabolites to zero. There is no obvious homologue of ThiH in *B. pertussis*. It was reasoned that the biosynthesis more closely resembles the pathway described in *B. subtilis* involving ThiS, ThiF and ThiG for which there are obvious homologs in *B. pertussis*(encoded by BP3690, BP0610 and BP3597 respectively) along with thiazole tautomerase, TenI (BP3809) and ThiE (BP0316). The model was curated to include this biosynthetic pathway.

### LPS biosynthesis

The SEED metabolic models include LPS biosynthesis based on the *E. coli* LPS structure. The structure of *B. pertussis* LPS is known, and the genetics of its biosynthesis is well-characterised [31–33]. Reactions for synthesis and assembly of the *B. pertussis* LPS molecule were substituted for the *E. coli*-based reactions, and the associated *B. pertussis* genes were assigned to these reactions. This involved modifying the reactants and products of two reactions, the addition of nine new reactions and removing thirteen of the *E. coli* LPS-specific reactions. LPS is most abundant molecule in the outer leaflet of the outer membrane of gram negative bacteria. Constructing an accurate *B. pertussis* LPS biomass component enhances the accuracy of the model.

### Freewheeling reactions

Several reactions involving electron transfer were set by ModelSEED to operate in the opposite direction to the thermodynamically feasible direction for electron transport, producing unfeasibly large fluxes at no energetic cost. The direction of these transfers was reversed, S3 Table.

### Tuning to known growth media

Previous studies have identified a number of carbon sources that either can or can not be metabolised by *B. pertussis* [3, 4, 8, 25]. Exchange reactions were modified to include the uptake of the metabolisable carbon sources, along with ammonia that can be used as a source of nitrogen by *B. pertussis*: pyruvate, L-aspartate, L-arginine’, L-alanine, L-glycine, L-histidine, 2-oxoglutarate, malate, L-lactate, ammonia.

### Blocked reactions/dead end metabolites

The requirement that all metabolites remain at a constant concentration is a central approximation in FBA, and this places a basic limit that all metabolites must appear at least twice in the model if they are to take an active part in any fluxes. As a direct consequence, any reaction that contains a singularly-appearing metabolite (a dead-end metabolite) has its flux constrained to zero, regardless of the state of the rest of the network. Removing these metabolites and reactions from the model entirely has no impact on the model’s results. Our curated *B. pertussis* model contains 301 singleton metabolites, which take part in a total of 199 reactions, consequently all blocked. Assuming the annotations and associated genes are correct, their presence points to further missing reactions, completing the pathways from which they come. Alternatively, these reactions are the remnants of pathways from which enzymes are missing due to the extensive gene loss that has been a feature of *B. pertussis* evolution [6]. This extensive gene loss may have produced an unusually high number of degraded pathways. In this scenario, the reactions may be occuring but be producing dead-end metabolites. Given this uncertainty, they have been left in the model, but indicated with the *note* annotation *blocked:True*.

### Tuning BOF using biomass composition measurements

The biomass composition of *B. pertussis* was measured using triplicate cultures (see Methods): as percentage of dry cell weight, 53.9 (+/- 2.7) protein, 5.5 (+/- 1.9) carbohydrate, 4 (+/- 0.5) DNA, 3.5 (+/- 0.5) RNA and 9.5 (+/- 1) lipids. The BOF was tuned to incorporate these proportions of macromolecules.

### Gene essentiality

Gene essentiality was determined using the TraDIS approach. Three independent transposon libraries containing 300 000–500 000 colonies each were constructed. Insertion indices were calculated for each genes as described previously [21] (see Methods). This identified 415 genes as essential for growth under these conditions. A further 26 genes were ambiguous in terms of their essentiality but were not classed as essential in these studies. However, only 11 of the ambiguous genes appear in the model (S4 Table). One (BP3151) is associated with a singleton metabolite and thus a blocked reaction, and six others are part of multigene complexes (ribosomes, NADH dehydrogenase, DNA replication) formed by other essential genes and thus are associated with essential pathways/reactions, resulting in just four reactions associated with ambiguously essential genes appearing in the model.

**Fig 1:**
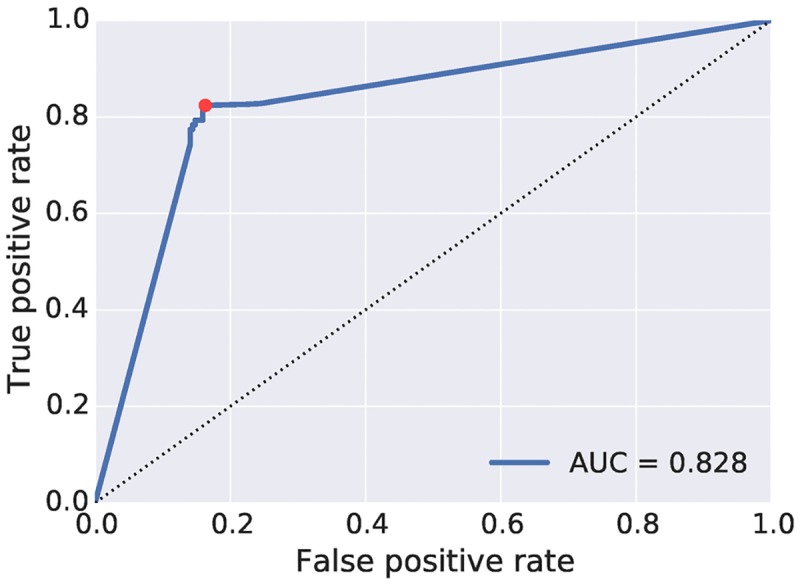
ROC curve showing performance of FBA essentiality predictions with variation of the the growth rate threshold. We treat the TraDIS results as the ground truth for essentiality of genes, and explore the prediction accuracy achieved by FBA simulation when the cutoff for simulated growth rate is varied.

Fig 1 shows ROC curves for FBA classification of gene essentiality, comparing model predictions of essentiality with experimentally defined essential genes. The AUC score demonstrates good classification. Fig 1 also shows as a red dot the selected threshold, chosen as the closest point to the perfect performance of (0,1).

In Table 2 we give the raw scores for the chosen threshold, divided into true and false positives and negatives. We present the results in a standard contingency table, identifying the types of errors made, as well as giving an overall accuracy score (calculated as (*TP* + *TN*)/(*TP* + *FP* + *TN* + *FN*)). The reactions for each of these categories are listed in S5 Table.

**Table 2:** Comparison of essentiality predictions with TraDIS results. TP/TN: true positive/true negative. FP/FN: false positive/false negative.

Curated Model	score = 0.83
TP = 226	FP = 92
FN = 44	TN = 430

When applying the FBA knockout approach to our network of metabolic reactions and associated genes, we achieve an accuracy of 83% in predicting the experimental essentiality. This compares well with scores achieved by other published metabolic models, and a perfect score is not to be expected, due both to experimental and theoretical considerations. While TraDIS is a state of the art approach, we cannot expect perfect results from TraDIS due to limitations in detecting extremely slow growing (but viable) mutants, and while our metabolic model reflects the current state of knowledge for *B. pertussis* metabolism, there remain uncharacterised proteins that may impact the performance of the network. Even accounting for errors in both TraDIS and the model, furthermore, FBA is an approach focused solely on the metabolic capabilities of an organism. There are regulatory and kinetic considerations that are beyond the scope of the FBA approach, but will nonetheless play a key role in the viability of knockout mutants. These considerations are likely to make perfect prediction an infeasible goal. Information on essential genes was used to refine some gene assignments for reactions. A number of reactions predicted to be essential had more than one possible gene assigned to them where it was not clear which gene was the correct assignment. In cases where one of the genes was shown to be essential, gene assignments were amended to show only this gene, as genes assigned to essential reactions also should be essential (S6 Table).

### Testing the model

**Fig 2:**
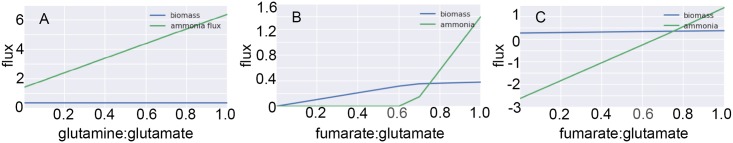
The flux of biomass production and ammonia production was modeled for *B. pertussis* growth using different ratios of (a) glutamate:glutamine, (b) glutamate:fumarate while constraining ammonia uptake and (c) glutamate:fumarate while allowing free uptake of ammonia. A glutamate:fumarate ratio of 1:2 (with regards to contribution of carbon atoms) was predicted to prevent production of ammonia while not affecting the growth rate.

A key use of metabolic models is to be able to make predictions of organism metabolism that can be investigated experimentally. To test our model, we sought to make predictions of changes to media formulations that decrease the production of growth inhibiting ammonia, without diminishing predicted growth rate. Ammonia production is thought to arise from an imbalanced N:C ratio when *B. pertussis* utilises glutamate as its sole carbon source [3]. To investigate this, we modelled the effect of shifting from growth on glutamate towards growth using glutamine (Fig 2a). Glutamine contains two amino groups compared to the one of glutamate. The model predicts that growth rate is unaffected whereas production of ammonia increases as the metabolism of glutamine over glutamte increases.

Next, we modelled the effect of metabolising different ratios of glutamate and fumarate (Fig 2b). Fumarate is an alternative carbon source but does not contain nitrogen. *B. pertussis* requires a nitrogen source to grow. If the uptake of ammonia as a source of nitrogen is prohibited then there is no growth in the model. However, as an increasing amount of glutamate is metabolised, with the corresponding decrease in fumarate metabolism, growth rate increases up to a point and the production of ammonia increases once a threshold ratio of glutamate:fumarate metabolised is reached. If this analysis is repeated allowing free uptake of ammonia, then the growth rate is unaffected by the ratio of glutamate:fumarate but ammonia is consumed up to a point when the metabolism of glutamate provides sufficient nitrogen, and ammonia is produced when the ratio of glutamate:fumarate metabolised reaches the point of imbalance between N:C (Fig 2c). This identified an approximate 1:2 ratio of glutamate to fumarate (in terms of contribution of carbon atoms rather than molecular mass) as an N:C balance at which ammonia production was minimised, but growth rate was unaffected, when the medium does not contain available ammonia.

**Fig 3:**
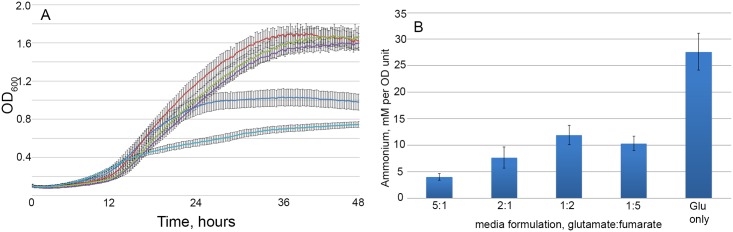
*B. pertussis* growth and production of ammonia in media with different glutamate:fumarate ratios. A) The growth of *B. pertussis* was monitored by measuring the increase in OD600 of cultures over time. The average OD of 10 replicate cultures is shown. B) Ammonia production was measured in the supernatants of 5 cultures for each medium at the end of growth. The average for each medium type is shown.

We tested this prediction experimentally by growing *B. pertussis* in different SS medium formulations in which carbon was provided by different ratios of glutamate:fumarate. The growth of *B. pertussis* was followed by measuring the absorbance of the culture (Fig 3a) and the concentration of ammonia was measured in cultures at the end point of growth (Fig 3b). Growth in media using solely glutamate as a carbon source resulted in relatively poor biomass yield and a relatively slow growth rate compared to media containing fumarate as a replacement for at least some of the glutamate. A glutamate:fumarate ratio of 5:1 produced moderate improvements in both rate and yield. Ratios of 2:1, 1:2 and 1:5 all gave dramatic improvements in rate and yield. The total amount of carbon in each medium was the same, suggesting that differences in biomass yields between cultures was most likely due to differing levels of inhibition of growth as opposed to nutrient limitation. Interestingly, replacement of some of the glutamate in the medium with fumarate resulted in a significant reduction in the level of ammonia produced by *B. pertussis*, on a ammonia per OD unit basis. A glutamate:fumarate ratio of 5:1 gave the greatest reduction while other ratios resulted in similar levels of ammonia. We suggest that the poor growth of the culture growing solely on glutamate was due to inhibition of growth by the resulting ammonia that was produced. The data demonstrate the model prediction to be largely correct in that balancing N:C ratios by the addition of fumarate reduced the production of ammonia, but that additional factors are evident as the growth of the cultures were clearly different from each other. This highlights the need for development of genome scale metabolic modeling to incorporate regulatory and non-metabolic constraints on growth.

## Discussion

We have developed and curated the first published genome-scale FBA model for *B. pertussis*, and have included an experimentally-determined biomass. The model predicts essential genes with 83% accuracy, compared with the state-of-the-art determination of essential genes with the TraDIS technique. The model and related computations are available in python in the pyabolism module. In contrast with our curated model, the automated SEED model based on the annotated *B. pertussis* genome cannot produce biomass on the standard growth medium for *B. pertussis* (SS broth). Extensive curation is typically required for genome-scale metabolic models [17], and in our case, this curation made fundamental differences to the model metabolism, enabling both growth on SS broth and accurate classification of essential genes.

While FBA models have extensive potential for applications, there are several remaining challenges. In particular, while genome annotation and function prediction are improving, the presence of genes classed as ‘hypothetical protein’ or with unknown function, and the presence of mis-classified genes, means that even with curation the accuracy of reconstructed models can be limited. This is a particular challenge for less-studied organisms; FBA models perform extremely well for well-characterized organisms such as *E. coli.* [20]. Even if the stoichiometric matrix were able to perfectly capture the metabolic reactions in an organism, there are reaction kinetics, regulatory interactions, the dynamics of transcription and translation and other important processes that are not captured in constraint-based models. Despite these limitations, the number of interesting applications in diverse micro-organisms has grown tremendously in recent years [34–38]. For this field to yield the results that have been promised, it is essential that the community develop and curate FBA models for more organisms—as we have done here.

*B. pertussis* presents some unique challenges and opportunities for constraint-based metabolic modeling. For example, *B. pertussis* evolved from its ancestor (*B. bronchiseptica*, or a *B. bronchiseptica*-like relative) by a process of genome reduction and rearrangement [6]. This has resulted in a large number of pseudogenes, which were not always recognised as being non-functional by the automated model construction. Also, gene loss has resulted in a number of incomplete, presumably remnant, metabolic pathways which automated gap filling attempts to ‘correct’ by adding missing functions, on the assumption that a pathway that was mostly present must be fully functional. The raw SEED model was unable to produce biomass when simulations were run using the components of the standard growth medium for *B. pertussis*, SS broth, as inputs. Thus, the production of a metabolic model that mimics the known characteristics of the organism required extensive and laborious manual curation.

*B. pertussis* is considered a re-emerging pathogen, with pertussis disease resurgent in numerous countries [39]. This has been associated with a change from the use of first generation, whole cell to second-generation, acellular pertussis vaccines. This resurgence has generated renewed interest in understanding the physiology and infection biology of *B. pertussis*. Understanding the basic growth of the bacterium is key to this, and a genome scale metabolic model is a widely applicable tool towards this goal. In addition, millions of doses of pertussis vaccines are used globally each year. An increase in demand for these vaccines, through either replacement of whole cell with acellular vaccines in more parts of the world, or expanded use of booster vaccinations to combat resurgence, will generate considerable strain on the global vaccine supply. Enhancement of the vaccine production process through shorter production times and increased yields from production will be important to meeting any increased demand. Understanding, and the ability to manipulate, *B. pertussis* growth characteristics is important towards this aim. The genome-scale metabolic model described here provides a novel tool to investigate *B. pertussis* growth and physiology. In particular, it allows the effects of altered medium formulations or genetic manipulation of metabolism to be investigated in silico, enabling much more targeted experimental investigations than are currently possible. The alteration of *B. pertussis* growth by substituting fumarate for some of the glutamate in standard media demonstrate the validity of this approach.

## Supporting information

S1 Data False negative predictions. (CSV)

S2 Data False positive predictions. (CSV)

S3 Data True negative predictions. (CSV)

S4 Data True positive predictions. (CSV)

S5 Data Curated SBML model. (XML)

S6 Data Python notebook. (IPYNB)

S1 Table Reactions in the model for which KEGG associtions were manually corrected. (TXT)

S2 Table Gap filled reactions without an associated gene remaining in the model. (TXT)

S3 Table Reactions for which directionality was constrained. (TXT)

S4 Table Ambiguously essential genes and the associated reactions contained in the model. (TXT)

S5 Table Essential gene predictions with associated reactions. These can also be queried directly in the SBML, for example by looking at all reactions associated with a specific gene. Genes are categorised as either True Positives, True Negatives, False Positives or False Negatives.
(XLSX)

S6 Table Reactions for which gene associations were amended based on essential gene data. (TXT)
